# Editorial: Electron-Microscopy-Based Tools for Imaging Cellular Circuits and Organisms

**DOI:** 10.3389/fncir.2019.00064

**Published:** 2019-10-11

**Authors:** Yoshiyuki Kubota

**Affiliations:** ^1^Division of Cerebral Circuitry, National Institute for Physiological Sciences, Okazaki, Japan; ^2^Department of Physiological Sciences, The Graduate University for Advanced Studies (SOKENDAI), Okazaki, Japan

**Keywords:** volume EM, segmentation (image processing), neural network, artificial intelligence, connectome, SSEM, synapse

Electron microscopy (EM)-based reconstruction of neuronal circuits from serial ultrathin sections was introduced more than three decades ago (White and Keller, 1987). Initially, all the steps were conducted manually, including cutting serial ultrathin sections using the ultramicrotome, image capturing with transmission electron microscope (TEM), and reconstruction using cardboard pieces of selected profiles of neural structures to provide the impression of depth. In the 1990s, computer software assisted reconstruction methods to make it more efficient were introduced (Harris et al., 1992; White et al., 1994). This reconstruction analysis software was used in a limited number of laboratories where good skills for obtaining serial ultrathin sections had been established and, thus, significant and valuable results were obtained. In general, however, this reconstruction technology was not popular because of a high demand on skills to obtain high quality serial ultrathin sections. In the early 2000s, a number of groups, with many represented in this special issue, started to conduct neural network analyses with reconstruction of serial sections by adapting new EM technologies such as focused ion beam-scanning electron microscopy (FIB-SEM; Knott et al., 2008), serial block-face electron microscopy (SBEM; Denk and Horstmann, 2004; Ohno et al., 2014), automated tape-collecting ultramicrotomy (ATUM) with SEM (Terasaki et al., 2013), transmission electron microscope camera array (TEMCA; Bock et al., 2011), and transmission-mode SEM (Kuwajima et al., 2013). These approaches have been modified and improved vigorously (Kubota et al., 2018a,b), and a large amount of noteworthy results were published in the last decade (Tomassy et al., 2014; Kasthuri et al., 2015; Lee et al., 2016; Villa et al., 2016; Schmidt et al., 2017; Takemura et al., 2017; Bae et al., 2018). The size of EM volume data sets has grown year by year, and it could be huge especially when data are obtained with high-throughput EM systems of either TEMCA (Bock et al., 2011; Lee et al., 2016), multi-beam SEM (Eberle and Zeidler; Shibata et al.) or parallel processing with multiple single beam SEM systems (Plaza and Funke; Scheffer.) For instance, a 100 cubic μm EM data set with 5 nm/pixel and 30 nm z-step of mouse cortex block, which amounts to 1.3 TB, was obtained that provides a sufficient resolution to detect synaptic contacts. This data set should contain about 1,000,000 synapses (Merchan-Perez et al., 2009) and be sufficient in volume to include many different kinds of connections among a wide variety of cortical neuron subtypes and afferent axonal fibers from other brain regions. Such large volume EM data sets could not be acquired with the conventional manually operated EM using the ultramicrotomes and TEM (White and Keller, 1987; Kubota and Kawaguchi, 2000; Kubota et al., 2015; Marc et al.).

The success of large volume EM acquisition using these new EM systems has created an issue, i.e., how to process large image data sets thus obtained. Soon it became obvious that it was difficult to handle large EM volume data sets using conventional 3D reconstruction image processing computer applications that had been developed for the conventional EM data sets obtained with the ultramicrotome and TEM. Therefore, new image processing tools that can handle large volume data sets have been developed. For example, NIH imageJ plugins provide useful tools to stitch tiles for montage and to align serial section images (Cardona et al., 2012). The current bottle neck is the segmentation process. Currently, the majority of researchers working on EM volume data pursue segmentation of their data obtained from brains of a wide variety of animal species: *Caenorhabditis elegans* (Mulcahy et al.), leech (Pipkin et al.), Drosophila (Takemura et al., 2017), Zebrafish (Wanner and Vishwanathan), mouse (Maclachlan et al.), rat, marmoset, and human, and use manual image processing applications including: VAST, Reconstruct, Knossos, and others (Fiala, 2005; Dorkenwald et al., 2017; Berger et al.). To achieve segmentation easily and efficiently, automated segmentation computer applications have been developed (Januszewski et al., 2018; Lee et al., 2019) and used for many EM volume data sets. Segmentation performance has increasingly been improved and achieved coverage of ~90% of the volume, but it is not yet perfect (Plaza and Funke). Annotators are used to fix segmentation errors to create correct wiring of brain networks. This can be done manually using image software with a proof-reading function (Zhao et al.; Katz et al.). Hopefully, segmentation performance will improve further in the near future to reach an almost 100% success rate while reducing the time required for the proof-reading process. Toward this goal, the histological process should be improved (Hua et al., 2015; Mikula and Denk, 2015; Mikula, 2016; Genoud et al.; Maclachlan et al.; Nguyen et al.) and image processing tools with better performance (Berger et al.; Jorstad et al.; Titze et al.) should be developed. Finally, despite these technological advances, analyzing fully segmented EM volume data sets can be done only manually by researchers so far, who must have a good knowledge and understanding of brain networks. In addition, automated cell type identification tool (Schubert et al., 2019), automated synapse detection tool (Staffler et al., 2017), and correlated light and electron microscopy methods (Kubota et al., 2015; Wanner and Vishwanathan) are useful for neural network analyses. This special issue covers most of the cutting-edge 3D-EM methods currently available.

On September 8th, 2017, I contacted Shawn Mikula at the Max Planck Institute in Martinsried, to ask him if he would be willing to work as co-editor with me on a special issue of Frontiers in Neural Circuits Research Topic “Volume electron microscopy for neuroscience.” I wanted Shawn to be my partner to edit the special issue because I knew that he had a deep knowledge not only for the EM volume data set analysis but also histology, chemistry, mathematics and other areas. He immediately accepted the invitation and we chose the title of the Research Topic “Electron-Microscopy-Based Tools for Imaging Cellular Circuits and Organisms.” We started to invite contributors to the special topic issue at the end of October, 2017. Shawn invited many excellent researchers who have been developing image analysis applications or systems and/or working on large volume EM data sets. His selections indeed led to the success of this special topic issue. Subsequently, Shawn joined my laboratory at the National Institute for Physiological Sciences in Okazaki, Japan, briefly from January 2nd to March 30th, 2018. After visiting his family in the USA in April and May of 2018, he moved to the Keio University School of Medicine in Tokyo. With great sadness and most unfortunately, on July 8th, 2018 we lost Shawn tragically, when we just started reviewing a few manuscripts submitted.

This special issue reflects Shawn Mikula's great interest in the brain network architecture and his commitment to introduce the best technology available to all researchers conducting neuroscience research with EM volume data set analyses. I took over all the editorial work after Shawn was lost and always kept these convictions with me during the editorial work. I hope he would appreciate the results. Finally, I express my sincerest condolences and special thanks to Shawn Mikula's family.

## Author Contributions

The author confirms being the sole contributor of this work and has approved it for publication.

### Conflict of Interest

The author declares that the research was conducted in the absence of any commercial or financial relationships that could be construed as a potential conflict of interest.

## References

[B1] Bae J. A. Mu S. Kim J. S. Turner N. L. Tartavull I. Kemnitz N.. (2018). Digital museum of retinal ganglion cells with dense anatomy and physiology. Cell 173, 1293–1306.e19. 10.1016/j.cell.2018.04.040 29775596 PMC6556895

[B2] Bock D. D. Lee W. C. Kerlin A. M. Andermann M. L. Hood G. Wetzel A. W.. (2011). Network anatomy and *in vivo* physiology of visual cortical neurons. Nature 471, 177–182. 10.1038/nature09802 21390124 PMC3095821

[B3] Cardona A. Saalfeld S. Schindelin J. Arganda-Carreras I. Preibisch S. Longair M.. (2012). TrakEM2 software for neural circuit reconstruction. PLoS ONE 7:e38011. 10.1371/journal.pone.0038011 22723842 PMC3378562

[B4] Denk W. Horstmann H. (2004). Serial block-face scanning electron microscopy to reconstruct three-dimensional tissue nanostructure. PLoS Biol. 2:e329. 10.1371/journal.pbio.0020329 15514700 PMC524270

[B5] Dorkenwald S. Schubert P. J. Killinger M. F. Urban G. Mikula S. Svara F.. (2017). Automated synaptic connectivity inference for volume electron microscopy. Nat. Methods 14, 435–442. 10.1038/nmeth.4206 28250467

[B6] Fiala J. C. (2005). Reconstruct: a free editor for serial section microscopy. J. Microsc. 218 (Pt 1), 52–61. 10.1111/j.1365-2818.2005.01466.x 15817063

[B7] Harris K. M. Jensen F. E. Tsao B. (1992). Three-dimensional structure of dendritic spines and synapses in rat hippocampus (CA1) at postnatal day 15 and adult ages: implications for the maturation of synaptic physiology and long-term potentiation. J. Neurosci. 12, 2685–2705. 1613552 10.1523/JNEUROSCI.12-07-02685.1992 PMC6575840

[B8] Hua Y. Laserstein P. Helmstaedter M. (2015). Large-volume en-bloc staining for electron microscopy-based connectomics. Nat. Commun. 6:7923. 10.1038/ncomms8923 26235643 PMC4532871

[B9] Januszewski M. Kornfeld J. Li P. H. Pope A. Blakely T. Lindsey L.. (2018). High-precision automated reconstruction of neurons with flood-filling networks. Nat. Methods 15, 605–610. 10.1038/s41592-018-0049-4 30013046

[B10] Kasthuri N. Hayworth K. J. Berger D. R. Schalek R. L. Conchello J. A. Knowles-Barley S.. (2015). Saturated reconstruction of a volume of neocortex. Cell 162, 648–661. 10.1016/j.cell.2015.06.054 26232230

[B11] Knott G. Marchman H. Wall D. Lich B. (2008). Serial section scanning electron microscopy of adult brain tissue using focused ion beam milling. J. Neurosci. 28, 2959–2964. 10.1523/JNEUROSCI.3189-07.2008 18353998 PMC6670719

[B12] Kubota Y. Kawaguchi Y. (2000). Dependence of GABAergic synaptic areas on the interneuron type and target size. J. Neurosci. 20, 375–386. 10.1523/JNEUROSCI.20-01-00375.2000 10627614 PMC6774130

[B13] Kubota Y. Kondo S. Nomura M. Hatada S. Yamaguchi N. Mohamed A. A.. (2015). Functional effects of distinct innervation styles of pyramidal cells by fast spiking cortical interneurons. Elife 4:07919. 10.7554/eLife.07919 26142457 PMC4518632

[B14] Kubota Y. Sohn J. Hatada S. Schurr M. Straehle J. Gour A.. (2018b). A carbon nanotube tape for serial-section electron microscopy of brain ultrastructure. Nat. Commun. 9:437. 10.1038/s41467-017-02768-7 29382816 PMC5789869

[B15] Kubota Y. Sohn J. Kawaguchi Y. (2018a). Large volume electron microscopy and neural microcircuit analysis. Front. Neural Circuits 12:98. 10.3389/fncir.2018.00098 30483066 PMC6240581

[B16] Kuwajima M. Mendenhall J. M. Lindsey L. F. Harris K. M. (2013). Automated transmission-mode scanning electron microscopy (tSEM) for large volume analysis at nanoscale resolution. PLoS ONE 8:e59573. 10.1371/journal.pone.0059573 23555711 PMC3608656

[B17] Lee K. Turner N. Macrina T. Wu J. Lu R. Seung H. S. (2019). Convolutional nets for reconstructing neural circuits from brain images acquired by serial section electron microscopy. Curr. Opin. Neurobiol. 55, 188–198. 10.1016/j.conb.2019.04.001 31071619 PMC6559369

[B18] Lee W. C. Bonin V. Reed M. Graham B. J. Hood G. Glattfelder K.. (2016). Anatomy and function of an excitatory network in the visual cortex. Nature 532, 370–374. 10.1038/nature17192 27018655 PMC4844839

[B19] Merchan-Perez A. Rodriguez J. R. Alonso-Nanclares L. Schertel A. Defelipe J. (2009). Counting synapses using FIB/SEM microscopy: a true revolution for ultrastructural volume reconstruction. Front. Neuroanat. 3:18. 10.3389/neuro.05.018.2009 19949485 PMC2784681

[B20] Mikula S. (2016). Progress towards mammalian whole-brain cellular connectomics. Front. Neuroanat. 10:62. 10.3389/fnana.2016.00062 27445704 PMC4927572

[B21] Mikula S. Denk W. (2015). High-resolution whole-brain staining for electron microscopic circuit reconstruction. Nat. Methods 12, 541–546. 10.1038/nmeth.3361 25867849

[B22] Ohno N. Chiang H. Mahad D. J. Kidd G. J. Liu L. Ransohoff R. M.. (2014). Mitochondrial immobilization mediated by syntaphilin facilitates survival of demyelinated axons. Proc. Natl. Acad. Sci. U.S.A. 111, 9953–9958. 10.1073/pnas.1401155111 24958879 PMC4103317

[B23] Schmidt H. Gour A. Straehle J. Boergens K. M. Brecht M. Helmstaedter M. (2017). Axonal synapse sorting in medial entorhinal cortex. Nature 549, 469–475. 10.1038/nature24005 28959971

[B24] Schubert P. J. Dorkenwald S. Januszewski M. Jain V. Kornfeld J. (2019). Learning cellular morphology with neural networks. Nat. Commun. 10:2736. 10.1038/s41467-019-10836-3 31227718 PMC6588634

[B25] Staffler B. Berning M. Boergens K. M. Gour A. Smagt P. V. Helmstaedter M. (2017). SynEM, automated synapse detection for connectomics. Elife 6:e26414. 10.7554/eLife.26414 28708060 PMC5658066

[B26] Takemura S. Y. Nern A. Chklovskii D. B. Scheffer L. K. Rubin G. M. Meinertzhagen I. A. (2017). The comprehensive connectome of a neural substrate for ‘ON’ motion detection in Drosophila. Elife 6:e24394. 10.7554/eLife.24394 28432786 PMC5435463

[B27] Terasaki M. Shemesh T. Kasthuri N. Klemm R. W. Schalek R. Hayworth K. J.. (2013). Stacked endoplasmic reticulum sheets are connected by helicoidal membrane motifs. Cell 154, 285–296. 10.1016/j.cell.2013.06.031 23870120 PMC3767119

[B28] Tomassy G. S. Berger D. R. Chen H. H. Kasthuri N. Hayworth K. J. Vercelli A.. (2014). Distinct profiles of myelin distribution along single axons of pyramidal neurons in the neocortex. Science 344, 319–324. 10.1126/science.1249766 24744380 PMC4122120

[B29] Villa K. L. Berry K. P. Subramanian J. Cha J. W. Oh W. C. Kwon H. B.. (2016). Inhibitory synapses are repeatedly assembled and removed at persistent sites *in vivo*. Neuron 89, 756–769. 10.1016/j.neuron.2016.01.010 26853302 PMC4760889

[B30] White E. L. Amitai Y. Gutnick M. J. (1994). A comparison of synapses onto the somata of intrinsically bursting and regular spiking neurons in layer V of rat SmI cortex. J. Comp. Neurol. 342, 1–14. 10.1002/cne.903420102 8207123

[B31] White E. L. Keller A. (1987). Intrinsic circuitry involving the local axon collaterals of corticothalamic projection cells in mouse SmI cortex. J. Comp. Neurol. 262, 13–26. 10.1002/cne.902620103 3624546

